# Dissecting the effects of androgen deprivation therapy on cadherin switching in advanced prostate cancer: A molecular perspective

**DOI:** 10.32604/or.2022.026074

**Published:** 2023-01-12

**Authors:** LOKMAN VARISLI, VEYSEL TOLAN, JIYAN H. CEN, SPIROS VLAHOPOULOS, OSMAN CEN

**Affiliations:** 1 Department of Molecular Biology and Genetics, Science Faculty, Dicle University, Diyarbakir, 21280, Turkey; 2 Cancer Research Center, Dicle University, Diyarbakir, 21280, Turkey; 3 Department of Chemical and Biomolecular Engineering, University of Illinois, Urbana, IL, 61801, USA; 4 First Department of Pediatrics, National and Kapodistrian University of Athens, Athens, 11527, Greece; 5 Department of Microbiology and Immunology, Feinberg School of Medicine, Northwestern University, Chicago, IL, 60611, USA; 6 Department of Natural Sciences and Engineering, John Wood College, Quincy, IL, 62305, USA

**Keywords:** Prostate cancer, Androgen signaling, Androgen deprivation therapy, Cadherin switching

## Abstract

Prostate cancer is one of the most often diagnosed malignancies in males and its prevalence is rising in both developed and developing countries. Androgen deprivation therapy has been used as a standard treatment approach for advanced prostate cancer for more than 80 years. The primary aim of androgen deprivation therapy is to decrease circulatory androgen and block androgen signaling. Although a partly remediation is accomplished at the beginning of treatment, some cell populations become refractory to androgen deprivation therapy and continue to metastasize. Recent evidences suggest that androgen deprivation therapy may cause cadherin switching, from E-cadherin to N-cadherin, which is the hallmark of epithelial-mesenchymal transition. Diverse direct and indirect mechanisms are involved in this switching and consequently, the cadherin pool changes from E-cadherin to N-cadherin in the epithelial cells. Since E-cadherin represses invasive and migrative behaviors of the tumor cells, the loss of E-cadherin disrupts epithelial tissue structure leading to the release of tumor cells into surrounding tissues and circulation. In this study, we review the androgen deprivation therapy-dependent cadherin switching in advanced prostate cancer with emphasis on its molecular basis especially the transcriptional factors regulated through TFG-β pathway.

## Introduction

Prostate cancer (PCa) is one of the most frequently diagnosed malignancies in males with an increasing prevalence in both developed and developing countries and is one of the major causes of cancer-related death in men [1,2]. Although the standard treatment for local PCa is surgery and radiotherapy, some other treatment protocols, such as brachytherapy and androgen deprivation therapy (ADT) especially in patients with advanced PCa are commonly used [3,4].

The primary purpose of ADT is to reduce circulating androgen levels and block androgen signaling, since androgen has an important role in cancer cell proliferation and consequently disease progression [5]. Indeed, ADT is a powerful and useful treatment approach that causes a decrease in prostate-specific antigen (PSA) levels and tumor volume. It provides a temporary relief to patients although some patients may experience severe to moderate side effects such as hot flashes and sexual dysfunction during treatment [6,7].

**Figure 1:**
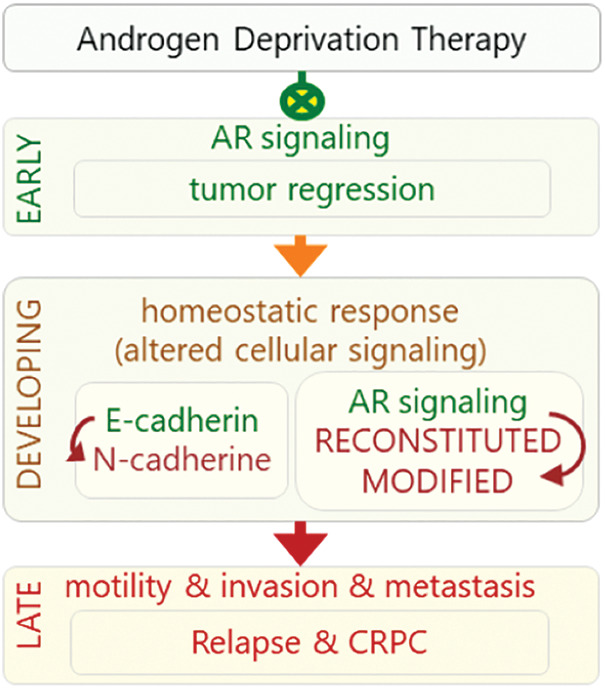
Response to ADT can be viewed in three phases: early, developing, and late phases. In early phase, ADT inhibits (green crossed-circles) AR signaling that leads to tumor regression. In the developing phase, cell’s homeostatic response results in re-activation of AR signaling in unconventional manners. Altered activities of signaling mechanisms lead to late phase (red arrow), in which castration resistance and more aggressive and highly metastatic AR-independent tumors develop.

The response to the ADT may be observed in three phases: early, developing, and late phases (Fig. 1). The early phase is characteristic with decreased level of PSA, tumor regression, and increased quality of life while the late phase is characteristic with castration resistance, relapse, and highly metastatic and aggressive tumors. In the intervening period, which can be considered as a developing phase, homeostatic response of the cells leads to a progressive development of alteration in various cellular signaling mechanisms leading to the late phase response (Fig. 1). The cellular responses include reactivation of androgen receptor (AR) signaling through alternative mechanisms, decreased adhesion, increased motility and invasion, and altered tonic cell survival signaling. Mechanisms involving re-activation of androgen signaling include intra-tumor *de novo* androgen production, amplification of *AR* gene, mutations in the ligand-binding domain of AR (AR-LBD), expression of some constitutively active AR isoforms, overexpression of AR co-regulators, and androgen-independent AR activation [8,9]. These events lead to a more aggressive phenotype of the disease called castration-resistant PCa (CRPC), generally 24–36 months after the initiation of ADT [10]. In this stage, metastatic spread is observed in most of the patients and the average life expectancy is 18–24 months afterward [10].

Another altered mechanism is epithelial-mesenchymal transition (EMT), which decreases adhesion and increases motility and invasion. Growing evidence links EMT to ADT in advanced PCa despite the lack of consensus regarding the effect of androgen signaling in the regulation of EMT [11]. Indeed, initial studies have reported that ADT may induce EMT in advanced PCa at least through the “cadherin switching” mechanism [12–18]. These reports led to further investigation, which pointed to an aberrant activation of transforming growth factor β1 (TGF-β) signaling [19,20], a multifunctional pathway regulating proliferation and differentiation of cells and cellular signaling molecules involved in cadherin switching [21]. The term “cadherin switching” generally refers to diminished expression of E-cadherin, encoded by *Cdh1*, and increased expression of N-cadherin, encoded by *Cdh2*, in cells [22]. Cadherin switching is a normal process during embryogenesis and cannot initiate tumorigenesis alone in healthy cells but may confer further migration and increased invasive capabilities on tumor cells [22–24]. Decreased *Cdh1* expression disrupts epithelial tissue structure causing cells to detach from one another and move freely [25]. Consistently, decreased expression of *Cdh1* depends on cadherin switching, a process closely associated with tumor invasion and metastasis, two common characteristics of advanced cancers [26]. N-cadherin confers enhanced migratory and invasive abilities on tumor cells, which contrasts the inhibitory role of E-cadherin on migration and invasion [27].

In this study, we aim to review the current literature to dissect the role of ADT on cadherin switching in advanced PCa from a molecular perspective.

## PCa and Androgen Signaling

PCa usually develops from pre-malignant lesions such as proliferative inflammatory atrophy (PIA) and prostatic intraepithelial neoplasia (PIN) to androgen-dependent local tumors and eventually progresses to CRPC [28]. The androgens are steroid hormones that play crucial roles in all steps of PCa from benign local tumors to malignant CRPC [29,30]. They are mainly synthesized in Leydig cells in testes through enzymatic conversion reactions from cholesterol to testosterone (T). Following synthesis, T is secreted into the blood where it is mostly carried as bound to serum albumin or serum hormone-binding globulin [31]. The circulating level of T is strictly regulated by the hypothalamus-pituitary–gonadal (HPG) axis feedback system [32]. T is a low potency hormone and after entering prostate cells it is converted to dihydrotestosterone (DHT) by 5-α-reductase [33]. Compared to T, DHT is more potent in activating AR signaling [33,34]. Although T can activate AR in prostate cells, conversion to DHT is necessary for the development of the normal prostate gland [35]. The necessity of DHT for the development of prostate has also been confirmed in the treatment of benign prostatic hyperplasia (BPH) by 5-α-reductase inhibitors [36,37].

**Figure 2:**
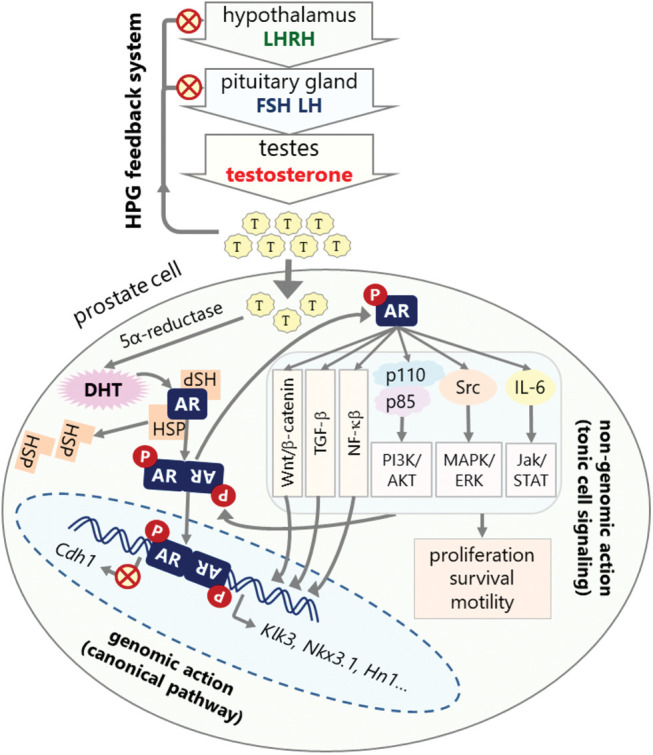
HPG axis feedback system for androgen synthesis and mechanism of androgen signaling. Androgens are mostly synthesized in testes under the control of the HPG feedback system. High androgen level inhibits hypothalamus and pituitary glands to decrease testosterone synthesis (red crossed-circles). Androgens exert their action in the prostate cells through canonical or non-genomic pathways. The canonical pathway involves transcriptional regulation of target genes. In this pathway, AR is sequestered in the cytoplasm by HSPs. Androgen binding activates and dimerizes AR, which is then translocated to the nucleus and binds to the AR elements in the promoters of target genes such as *Klk3*, *Nkx3.1*, *Hn1*, and *Cdh1* leading to the upregulation or downregulation of their expressions depending on the presence of other regulatory factors. Non-genomic action involves the direct activation of cellular signaling pathways, in which activated AR interacts with and phosphorylates (P in red circles) the components of various signaling pathways such as PI3K/AKT, MAPK/Raf/ERK, Jak/STAT, TGF-β, NF-κB, and Wnt/β-catenin pathways leading to the activation of these pathways that promote survival, proliferation, and metastasis of cell (red crossed-circles: inhibition, gray arrows: activation and interaction).

The action of androgen is mainly through AR [38]. In the absence of androgens, AR is mostly located in the cytoplasm, forming a heterocomplex with heat shock proteins (HSPs) comprised of HSP27, HSP40, HSP56, HSP70, and HSP90 [39,40]. Following ligand binding, AR can activate various metabolic cellular responses at genomic and non-genomic levels. At the genomic level, also known as the canonical pathway, activated AR leaves the HSP complex, dimerizes, and then is translocated to the nucleus (Fig. 2) [41]. In the nucleus, AR dimers bind to androgen response elements (ARE) on the promotors of the target genes, such as kallikrein related peptidase 3 (*Klk3*), NK3 homeobox 1 (*Nkx3.1*), hematological and neurological expressed sequence 1 (*Hn1*), and *Cdh1*, a process that controls the expression of these genes through recruiting various co-regulators such as transcriptional activators or suppressors [42–46].

At the non-genomic level, AR regulates diverse tonic cellular signaling mechanisms independent from AR nuclear transactivation (Fig. 2) [47,48]. One of the cellular kinases activated through AR signaling is Src kinase [49], which is highly active in PCa and is involved in increasing malignant behaviors of the tumor cells [49,50]. Phosphorylated Src is activated and can activate the downstream MAPK/ERK1-2 signaling pathway resulting in increased cell proliferation [49]. PI3K/AKT signaling pathway is also activated by AR in a non-genomic manner [51]. Activated AR interacts with the SH2 domain of the regulatory subunit (p85) of phosphatidylinositol 3-kinase (PI3K) in the cytoplasm, which promotes activation of the catalytic subunit (p110) of PI3K [52]. Consequently, PI3K activates serine/threonine kinase (AKT), a key molecule in the induction and maintenance of cell activation and survival signals. AR is also activated by Jak/STAT signaling pathway through IL-6 and possibly other cytokines [53–55]. MAP kinase and cytokine signaling pathways also activates the nuclear factor “kappa-light-chain-enhancer” of activated B-cells (NF-κB) pathway, which has been implicated in various cancers including prostate cancer. NF-κB is highly active in PCa that leads to increased AR expression and progression into androgen independent growth and CRPC [56,57]. Another deregulated pathway in PCa is Wnt/β-catenin signaling pathway, which is strictly regulated in normal cells. However, it has been dysregulated in various cancers including prostate cancer. While Wnt/β-catenin is inactive in early PCa, it is highly activated in late stages [58,59].

Overactivation of aforementioned signaling pathways and likely others, promote AR transactivation in a ligand independent manner via regulating interactions between AR and various co-regulators [51,53,54,60]. Consequently, this alternative activation of AR further activates cell proliferation and survival signaling, forming a feed-forward activation cycle [54]. Androgen-independent AR activation is a commonly observed mechanism in CRPC, which is further discussed below.

## ADT in the Treatment of Advanced PCa

While the treatment of choice for local PCa is usually surgery, radiotherapy, or brachytherapy, these treatments are often replaced with ADT in patients with advanced recurring PCa after prostatectomy [3,4]. The primary aim of ADT is to reduce circulating androgen levels and therefore inhibits or attenuate AR signaling [61]. The newly developed some third generation agents, such as enzalutamide, act as competitive androgen inhibitors by binding to androgen binding site of AR and inhibiting its androgen-induced transactivation [61]. It is estimated that about 40% of PCa patients will receive ADT within six months of diagnosis [5,62]. Indeed, ADT was proposed about 80 years ago to be a powerful treatment option for advanced PCa [63]. Although the original form of ADT was bilateral orchiectomy this procedure has later been replaced with certain drugs that block androgen production and AR signaling in PCa cells [4]. Currently, a combination of luteinizing hormone-releasing hormone (LHRH) agonists and anti-androgen agents are used to block androgen production [64].

At the beginning of treatment, ADT has a strong effect on the inhibition of the progression of the disease and leads to partial remediation such as regression in tumor volume and decreased serum PSA level, but this effect is temporary and some cell populations become refractory to ADT after a while [6]. Consequently, the disease begins progressing with an increase in the tumor volume and serum PSA level, leading to metastasis into new sites 24–36 months after ADT started [10]. The new phenotype of PCa is characteristic with stem cell-like features [65] and referred to as CRPC, which is associated with a poor prognosis [6,66]. The elevated serum PSA level is an important indicator of restored AR activity because activated AR directly promotes the transcription of PSA encoding gene *Klk3* [67].

Multiple AR re-activation mechanisms occur in CRPC including amplification of AR gene, the gain of function mutations in the AR-LBD, intra-tumoral *de novo* androgen production, increased transcriptional activation of AR, altered expressions of some AR co-regulators, growth factors- or cytokine-dependent AR activation, and expression of some constitutively active AR isoforms [68–74]. These mechanisms lead to AR stabilization and unconventional re-activation of AR signaling that ends up with increased transcriptional activity in CRPC even when there is a low androgen level.

AR gene amplification is the most common genetic change in CRPC patients. It has been shown that 20%–30% of the patients harbor amplified AR in their genomes in CRPC cells [75–77]. However, this amplification is not common in primary tumors and is reported in just 2% of PCa patients who had not undergone ADT [78]. Another cause of increased AR signaling is due to increased level of AR protein via post-transcriptional or post-translational deregulation, which may lead to increased stabilization of AR mRNA and/or protein [79,80]. Consequently, the increased level of AR protein in CRPC cells enables the cells to be hypersensitive to the low level of androgens [79,81,82].

Another mechanism for the re-activation of AR signaling in CRPC is AR mutations. While some mutations have been identified in the amino-terminal (NTD) and DNA-binding (DBD) domains of AR, most of the activating mutations are localized to the ligand-binding domain (LBD) of AR [79,83]. Although mutations in LBD are rare in hormone-responsive PCa cells, they have been detected in about 20% of CRPC tumor cells [84]. Mutations in LBD alters the ligand specificity of AR and facilitate its activation with other steroid hormones, in the absence of androgen [70,79]. Furthermore, it has been shown that anti-androgens may act as agonist on some AR with mutated LBD [83].

Although ADT largely suppresses the serum T level, intra-tumoral DHT level may still be high [85]. Intra-tumoral androgen production may be regulated by various mechanisms including *de novo* production of DHT and conversion of adrenal androgens or cholesterol to T [71,86,87]. Consequently, insufficient suppression of intra-tumoral androgens causes continued activation of AR signaling in the tumor cells even in castrate conditions [88].

Expression of constitutively active AR variants is an important mechanism that drive castration sensitive cells to CRPC [89,90]. These variants are expressed at later stages of PCa as a response to low androgen environment under ADT conditions [91,92]. Although more than 20 AR splice variants are expressed in PCa cells, the constitutively active variants AR-V3, AR-V4, AR-V7 and AR-V12 have been shown to be important in reconstituting the AR function [93,94]. Among these variants, the AR-V7 is the most extensively characterized AR variants [95,96]. AR7 mutant has truncated LBD due to the aberrant splicing events and its constitutive activity is ligand independent [95]. Lack of LBD in the AR-V7 renders AR-7 resistant to anti-androgens such as enzalutamide and abiraterone in CRPC cells. Therefore, AR-V7 is considered as a CRPC-specific AR variant [97–100]. AR-V7 is also associated with worse prognosis in PCa as the presence of AR-V7 is associated with poor survival in metastatic CRPC patients [89].

AR functions as a transcription factor in canonical signaling pathway and over 250 co-regulators (co-activators or co-repressors) have been shown to contribute to the regulation of AR transcriptional activity [101,102]. The term AR co-regulators represents a wide group of molecules that include proteins and RNAs that regulate AR activity [72] and the relationship between AR and co-regulators affects development, progression, and treatment outcome of PCa [103]. Indeed, AR co-regulators are differentially expressed in all stages of PCa including CRPC [104]. Furthermore, it has been suggested that differential expressions of AR co-regulators contribute to the development of CRPC [105] and altered levels of co-activators and co-repressors result in induction of AR transcriptional activity in CRPC, even at extremely low concentrations of androgen [104].

Growth factors and cytokines have been shown to activate AR even in the absence of or in the presence of low androgen concentration. For example, insulin-like growth factor-1 (IGF-1), epidermal growth factor (EGF) and keratinocyte growth factor can activate AR in androgen deprived conditions [73]. On the other hand, interleukin-6 (IL-6) can activate AR via Stat3, PI3K/AKT, and MAPK signaling in PCa cells [106,107]. Similarly, NF-κB induces increased AR transcription and protein [108] and promote androgen independent growth of cancer cells [56]. IL-8 can also induce androgen independent activation of AR in a FAK and Src dependent manner [109].

### Activation of β-catenin in PCa after ADT

Wnt/β-catenin signaling is a conserved pathway that regulates various physiological functions including differentiation, embryonic development, adhesion, migration, apoptosis, and tissue homeostasis. Binding of Wnt to its G-protein coupled receptor activates a signaling cascade leading to the accumulation and translocation of β-catenin into the nucleus where it recruits transcription factors leading the transcription of target genes. The Wnt/β-catenin signaling is strictly regulated in normal cells; however, it has been dysregulated in various cancers including prostate cancer [58,59]. One of the EMT-promoting factor Cripto-1 (CR-1), a member of epidermal growth factor family, is upregulated in metastatic prostate cancers and is associated with decreased membrane associated β-catenin and increased nuclear β-catenin [110,111] and knocking down its expression inhibits cell proliferation, migration, and invasion [112]. CR-1 has a central role in regulating and cross-talking with various cellular signaling mechanisms including TGF-β, MAPK, and Wnt/β-catenin [113]. Overexpressed CR-1 is a poor prognostic indicator in prostate cancer [114].

In addition, a number of microRNAs (miRNAs) have been shown to modulate Wnt/β-catenin pathway in cancer cells [115,116]. For example, miRNA miR-744 promotes prostate cancer progression through aberrant activation of Wnt/β-catenin pathway [117] while miR-15A, miR-138, and miR-320 suppresses Wnt/β-catenin pathway [118–120].

### TGF-β in PCa development and metastasis after ADT

TGF-β signaling is a multifunctional pathway regulating various cellular functions including embryonic development, tissue repair, differentiation, inflammation, migration, proliferation, and cell cycle [121]. TGF-β exerts its signaling through its heterodimeric receptors TβRI and TβRII leading to activation of its canonical (SMADs—transcriptional regulation) and non-canonical (TRAFs, PI3K/Akt/mTOR, and CDC42—regulation of tonic) cellular signaling mechanisms [122]. Aberrations in TGF-β signaling have been indicated in various diseases including cancers [21]. In PCa, TGF-β signaling has a dual role: while it represses survival signaling and promotes cell cycle arrest and pro-apoptotic signals in the normal prostate cells, it promotes oncogenic signals and treatment-resistant phenotypes in malignant and advanced tumor cells [123–125] [126]. TGF-β inhibits cell cycle progression in early-stage PCa cells, and TGF-β treatment together with androgen leads to a strong cell cycle arrest and apoptotic induction in the androgen-dependent PCa cells, it promotes invasion, angiogenesis, and metastasis in advanced PCa [127–129]. This contradicting effect may be related to the structure of its promoter, at least in the context of androgen signaling. The *Tgf-β1* promoter has multiple AREs that group into positive and negative AREs that respectively recruit positive and negative regulatory factors, and binding of AR to these elements regulates *Tgf-β1* expression positively or negatively depending on the combination of other regulatory factors [73]. In the normal prostate gland cells, activated AR binds to negatively regulating AREs on the TGF*-β1* promoter and inhibits its expression [130]. However, in the metastatic PCa cells, androgen stimulation leads to the binding of activated AR to positively regulating AREs on the *Tgf-β1* promoter and increases its expression [131]. *Tgf-β1* expression is consistently high in the metastatic PCa cells and elevated TGF-β1 level increases Bcl-2 level rendering cells resistant to chemotherapeutic agent Docetaxel [132,133].

The effect of ADT on the TGF-β signaling has also been investigated. Interestingly, it has been shown that ADT causes an increase in the levels of TGF-β ligand, TGF-β receptor I (TβRI), TGF-β receptor II (TβRII), and further activation of transcription factor SMAD3 [134–136]. It is likely that ADT may also regulate mRNA and protein levels of TGF-β1 by post-transcriptional mechanisms [137]. Another mechanism has also been proposed for androgen and ADT-dependent TGF-β1 regulation during PCa progression. This suggests that TGF-β1 regulation may be through SAM Pointed Domain Containing ETS Transcription Factor (SPDEF) that inhibits EMT and thereby metastatic behaviors of PCa cells, as described below [138].

## Cadherin Switching in the Progression of PCa and Its Relationship with Androgen Signaling

Cadherin switching usually refers to repression of *Cdh1* expression, which encodes for E-cadherin, and induction of *Cdh2* expression, which encodes for N-cadherin, and as a result, E-cadherin level decreases while N-cadherin level increases in the cells. E-cadherin is an important adhesion molecule for the formation and stability of epithelial tissue. Its loss impairs prostatic glandular formation and induces PIN in normal prostate luminal cells [139]. *Cdh1* expression is inversely regulated by androgen signaling [140]. Increased androgen activity suppresses *Cdh1* expression, which leads to decreased E-cadherin level, and consequently weaker cell-cell adhesion in androgen-dependent PCa cells [141]. Since ADT inhibits androgen signaling, the level of E-cadherin would be expected to increase upon ADT. In contrast, *Cdh1* expression is further inhibited in ADT, in an AR-independent manner via the activities of a group of transcription factors and other proteins that induce cadherin switching [142,143]. The loss of *Cdh1* expression impairs adhesion of primary tumor cells in many cancers, including PCa [15,27,144–146]. The association between decreased E-cadherin levels and metastasis has been shown in prostate tumors [147]. Furthermore, loss of E-cadherin in PCa has been shown to cause its metastasis into lymph nodes and bones, indication of poor prognosis [148–150].

N-cadherin, like E-cadherin, is a calcium-dependent adhesion molecule but it has opposite roles in tumorigenesis [151]. *Cdh2* is highly expressed in mesenchymal and neural cells while its expression is absent or at a very low level in epithelial cells [145,152]. However, its level is increased in many advanced tumors, including PCa, and is associated with the progression of the disease [27,153]. Indeed, N-cadherin level is increased in CRPC cells compared to the castration sensitive PCa cells [14]. Likewise, increased *Cdh2* expression causes castration resistance in PCa cells and targeting N-cadherin with monoclonal antibody inhibits the invasive and metastatic behaviors of the cells and renders them castration sensitive [14,153]. It is known that elevated *Cdh2* expression increases migrative and invasive abilities of cancer cells [27]. Indeed, it has been shown that N-cadherin induces EMT and thereby increases the metastatic potential of PCa cells [154].

**Figure 3:**
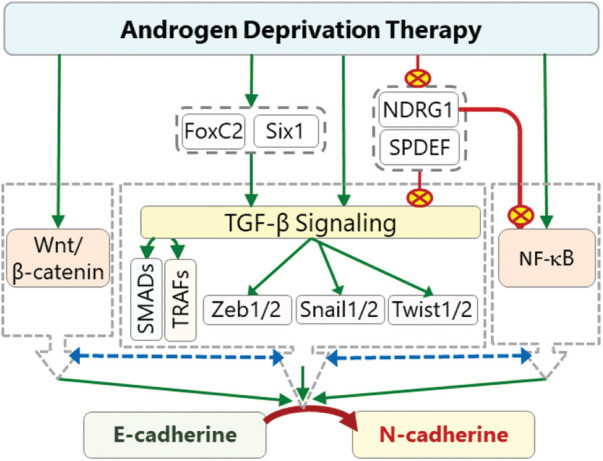
The effects of ADT on cadherin switching. ADT can activate TGF-β signaling directly or indirectly through activation (green arrows) of Six1 and inhibition (red crossed-circles) of SPDEF and NDRG1. Activated TGF-β induces the expression of *Zeb1*, *Twist*, and *Snail* transcription factors through SMADs (canonical) and TRAFs (non-canonical). These transcription factors promote cadherin switching by inhibiting *Cdh1* expression and activating *Cdh2* expression. Some of these factors can also induce the expression of *FoxC2* which also promote cadherin switching. ADT-dependent activated *Six1* can also directly or indirectly contribute to cadherin switching. ADT-induced repression of SPDEF and NDRG1 indirectly causes further increase in the ZEB1, TWIST, and SNAIL levels, and consequently results in a further decrease in *Cdh1* and increase in *Cdh2* expressions. The interaction (dashed two-way blue arrows) of TGF-β pathway with NF-κB and Wnt/β-catenin pathways further increase the intensity of cadherin switching (red crossed-circle: inhibition, green arrows: activation, dashed two-way blue arrows: interaction).

Androgen signaling has an important regulatory function on the level of N-cadherin in PCa cells. Indeed, it has been shown that androgen treatment causes a decrease in the N-cadherin level and inhibition of AR signaling upregulates N-cadherin expression and induces progression of PCa [12,16]. High *Cdh2* expression after ADT is also associated with a higher Gleason score and metastasis [12,155,156]. The data suggest that ADT may cause cadherin switching in PCa, which switches the cell growth from androgen-dependent state to androgen-independent state and consequently increases migrative and metastatic abilities of the cells [15,16,155]. Although many signaling mechanisms may play roles in the cadherin switching, TGF-β signaling has a key role in this transition (Fig. 3) [21,157]. Depending on cellular micromilieu, it may act as a strong EMT promoter, which leads to loss of cellular polarity and tight junctions due to decreased expression of E-cadherin and increased expression of N-cadherin that renders a highly migratory phenotype on the cells [21]. TGF-β pathway also crosstalk with NF-κβ and Wnt/β-catenin pathways in EMT modulation. The interaction involves use of common signaling molecules and transcription factors. For example, CR-1 induces EMT through activating Wnt/β-catenin pathway as well as SMADs in the canonical TGF-β signaling [113]. Likewise, CR-1 can activate SNAIL1 through Src/Akt axis [158].

TGF-β-mediated EMT also involves microRNAs in regulating transcriptional factors involving cadherin switching. TGF-β downregulates miR-200 family of microRNAs, which normally repress the ZEB1/2, but their downregulation by TGF-β leads to indirect increase in the level of ZEB1/2 [159]. Likewise, miR-34 and miR-1 microRNAs repress expression of SNAIL1 and SNAIL2, respectively, and downregulation of these microRNAs by TGF-β leads to their increased expression [160,161]. Both ZEBs and SNAILs promote cadherin switching. ZEB and SNAIL proteins also downregulate the expression of these microRNAs further promoting their own expression and amplifying the cadherin switching mechanism [21]. A long list of microRNAs beyond the scope of this review has been indicated in the PCa–CRPC transition but we have focused on those important in connecting TGF-β with the EMT. In addition to those mediating TGF-β-induced EMT, various miRNAs have been implicated in modulating the EMT through NF-κβ and Wnt/β-catenin pathways. For example, NF-κβ-induced miR-230 promotes EMT and bone metastasis of advanced prostate cancer [162]. miR-96-5p inhibits NDRG1, which inhibits NF-κB [163].

Although many direct and indirect transcriptional targets of TGF-β signaling have been identified in this process, Zeb1, Twist1, Snail1, Snail2, FoxC2 and Six1 play important roles and worth further discussion in the cadherin switching process [21]. Indeed, increased expressions of these factors have been shown to lead to cadherin switching in various cancers, including PCa [164–171]. The major factors involved in the ADT-induced cadherin switching are listed in Table 1 and discussed below.

**Table 1:** A limited list of prominent factors that induce cadherin switching discussed in this review

Gene	Cellular function	Expression under ADT	Effect on cadherin switching	Mechanism of action in cadherin switching	References
Zeb1	Transcription factor	Increases	Inducer	Direct and indirect regulation of E-cadherin/N-cadherin levels	[164,177,181–185]
Twist1	Transcription factor	Increases	Inducer	Direct and indirect regulation of E-cadherin/N-cadherin levels	[166,210,211]
Snail1/Snalil2	Transcription factor	Increases	Inducer	Direct and indirect regulation of E-cadherin N-cadherin levels	[165,169,229]
FoxC2	Transcription factor	Increases	Inducer	Indirectly inhibits E-cadherin expression—Directly increases N-cadherin levels.	[237,240,241]
Six1	Transcription factor	Increases	Inducer	Indirect regulation of E-cadherin/N-cadherin levels via increasing TGF-β signaling.	[246–248]
SPDEF	Transcription factor	Decreases	Repressor	Direct and indirect regulation of E-cadherin/N-cadherin levels	[138,255–257]
NDRG1	Cytoplasmic protein	Decreases	Repressor	Indirect regulation of E-cadherin/N-cadherin levels via inhibiting TGF-β signaling	[266,267]

### Zinc finger e-box binding homeobox 1 (Zeb1)

*Zeb1* has seminal roles in embryogenesis as *Zeb1* null mice die due to multiple lethal defects [172]. Altered expression of *Zeb1* has been reported in many cancers including PCa, and elevated *Zeb1* expression confers further malignant behaviors on the tumor cells [173–175]. In concordance, silencing *Zeb1* inhibits proliferation, migration, and invasion of Pca cells [174,176].

ZEB1 protein is a strong EMT inducer. Increased *Zeb1* gene expression induced by TGF-β, IGF-I or hypoxia promotes EMT in Pca cells [177,178], which is associated with poor prognosis [174,175]. In concordance, increased ZEB1 level has been shown to inhibit the epithelial phenotype of prostate cells and thereby increase their transendothelial migration [179]. ZEB1 level is also associated with vasculogenic mimicry and elevated ZEB1 is linked to a higher Gleason score and lymph node metastasis in Pca cells [180]. ZEB1 may influence the migrative and invasive behaviors of cells in multiple ways. The best known and probably the dominant mechanism is direct inhibition of *Cdh1* expression. In this mechanism, ZEB1 directly binds to the *Cdh1* promoter and recruits histone deacetylases HDAC1/HDAC2 and co-repressors to the *Cdh1* promoter and consequently represses its expression [164,181–184]. ZEB1 may also contribute to epigenetic silencing of Cdh1 by recruiting DNA methyltransferase 1 (DNMT1) to *Cdh1* promoter [185]. In addition, suppression of *Zeb1* expression reverses the suppression of E-cadherin and upregulation of N-cadherin protein levels in Pca cells [177].

ZEB1 may also influence the expression of other cell adhesion molecules. For example, ZEB1 represses the expression of Syndecan 1 (SDC-1), a cell surface proteoglycan that has roles in cell adhesion [186]. It also represses the expression of glycosyl transferase LARGE2 and thereby causes hypo-glycosylation of cell surface glycoprotein alpha dystroglycan (αDG), which leads to increased invasion and metastasis [187].

High ZEB1 level is also associated with chemotherapy and radiotherapy resistance of cancer cells [188–190], ZEB1 level is higher in docetaxel-resistant cells compared to sensitive cells, and silencing of *Zeb1* in docetaxel-resistant cells renders them docetaxel-sensitive [191,192]. Furthermore, it has been shown that ZEB1 may induce cancer stem cell features in various cancers and silencing of *Zeb1* decreases treatment resistance and stem cell-like characteristics in Pca [193,194].

There is a negative correlation between AR activity and ZEB1 level in Pca cells. Indeed, inhibition of androgen signaling using enzalutamide has been shown to cause an increase in the ZEB1 level in Pca cells [195]. In concordance, ZEB1 level is also increased in androgen dependent LNCaP Pca cells when cultured in androgen deprived conditions [16]. Although it is controversial whether *Zeb1* is a direct target of AR under physiological conditions, it is clear that AR and ZEB1 reciprocally regulate each other’s levels and activities in androgen-sensitive cells [196–199]. ZEB1 directly binds to AR promoter [199,200] and the importance of this binding in the presence or absence of androgen in prostate cells needs further investigation. However, it is clear that high ZEB1 promotes androgen independence via induction of stem cell-like properties while silencing *Zeb1* renders Pca cells sensitive to androgen [201].

Enzalutamide treatment may induce neurodifferentiation of Pca cells by activating a positive feedback loop between ZEB1 and the calcium-sensitive potassium channel SK3 [202]. Furthermore, this feedback loop between ZEB1 and SK3 promotes calcium entry and cellular migration of Pca cells [178,203].

### Twist family BHLH transcription factor 1 (Twist1)

Twist1 is an important transcription factor in health and cancer. It is important in EMT both during embryonic development and in cancer progression [168,204]. Although Twist1 exerts multiple effects in all stages of carcinogenesis including initiation, invasion, metastasis, angiogenesis, and therapy resistance, here, we focus only on the role of Twist1 in the cadherin switching mechanism [205,206]. The relationship between the cellular Twist1 level and PCa progression is relatively well documented [205]. It has been shown that *Twist1* expression is higher in PCa compared to normal prostate and there is a positive correlation between *Twist1* expression level and Gleason score in PCa [207]. Consistently, increased *Twist1* expression is associated with metastasis, and silencing of Twist1 represses cell invasiveness in androgen independent PCa cells [208,209]. TWIST1 can directly bind to both *Cdh1* promoter and the first intron of *Cdh2* [210] and regulate their expression resulting in decreased E-cadherin and increased N cadherin protein levels in the cells [166,210]. Twist1 can also represses *Cdh2* expression, indirectly, through inducing the expressions of other cadherin switching transcription factors, such as Snail 2 [211]. Furthermore, elevated Twist1 level is implicated in bone metastasis in PCa, an indication of poor prognosis [208].

*Twist1* expression is negatively regulated by androgen and inhibition of androgen signaling by enzalutamide causes an increase in the *Twist1* expression [195,212,213]. Accordingly, *Twist1* expression increases following ADT [16,214,215]. This regulation of Twist1 by AR is indirect as AR does not seem to directly bind to its promotor [212,213]. Indeed, although an early study has proposed that this indirect regulation is through NKX3.1, which is direct target of AR, a recent study has proposed that this regulation is mediated by ETS variant 1 (ETV1), another direct target of AR [212,213]. Furthermore, the effects of androgen on *Twist1* promoter activity may be activatory or inhibitory depending on the recruited co-regulators with ETV1 [213]. Shiota et al. [215] has reported that AR and TWIST1 reciprocally control the expressions of each other, and castration-induced oxidative stress may promote AR overexpression through the increase of *Twist1* expression leading to the development of castration resistance [215]. Interestingly, it was shown that inhibition of AR signaling induced *Twist1* expression via PKC activation, which led to castration resistance through upregulation of AR [216]. In this mechanism, NF-κB signaling has been shown to be responsible for PKC-dependent increased Twist1 expression [217]. This association may be a critical node in explaining *Twist1*-dependent PCa growth in androgen-dependent state.

### Snail family transcriptional repressor 1 (Snail1) and Snail2

Increased expressions of *Snail1* (*Snail*) and *Snail2* (*Slug*) have been reported in various cancers and their elevated expressions are associated with further motility, invasiveness, and metastasis of cancer cells [195,218–221]. Concordantly, both *Snail1* and *Snail2* are overexpressed in PCa, which is associated with a higher Gleason score [222–224]. Furthermore, silencing of *Snail1* or *Snail2* represses the migrative and invasive abilities of PCa cells [218,225]. In fact, the SNAIL proteins inhibit the expression of not only *Cdh1* but also many other genes associated with the epithelial structure and function such as claudins, occludin, and other components of tight junctions [226]. On the other hand, they activate the genes of mesenchymal markers including *Cdh2*, vimentin (*Vim*), and matrix metalloproteinases (*MMP*s) [227–229]. Indeed, it has been shown that both Snail1 and Snail2 directly bind to *Cdh1* promoter and represses its expression [165,169].

The expressions of both *Snail1* and *Snail2* are regulated by androgens [195,230]. Enzalutamide-mediated inhibition of androgen signaling leads to an increase in the expression of *Snail1*, and this increased expression promotes resistance to ADT [195,231]. Similarly, elevated *Snail2* expression confers a growth advantage on androgen-deprived PCa cells, and the loss of *Snail2* expression correlates with a better clinical response to ADT [230,232]. On the other hand, *Snail2* overexpression causes an increase in the AR protein level, with which it forms a complex to cooperatively regulate downstream gene expression leading to castration resistance [230].

### Forkhead box protein C2 (FoxC2)

FOXC2 is a member of FOX family transcription factors, and its increased expression is associated with metastasis and poor prognosis in various cancers, including PCa [233–235]. Although it is not known whether androgen directly regulates *FoxC2* expression, inhibition of androgen signaling by enzalutamide causes increased *FoxC2* expression [195,236]. On the other hand, it has been shown that increased FOXC2 inhibits AR expression in a *Zeb1*-dependent manner, thereby contributing to castration-resistance [237]. Increased FOXC2 level has been shown to cause a decrease in the E-cadherin and an increase in the N-cadherin levels [237]. However, direct binding of FOXC2 to E-cadherin promoter could not be demonstrated and therefore the negative effect of FOXC2 on E-cadherin level is likely indirect. However, FOXC2 has been shown to directly bind to the promoter of p120-catenin, whose protein product β-catenin protects E-cadherin from endocytosis and proteasomal degradation [238,239]. Furthermore, increased FOXC2 protein level causes an increase in the ZEB1 and SNAIL protein levels [237]. Indeed, it was shown that FOXC2 can bind to *Zeb1* promoter when phosphorylated on serine 367 by p38 kinase [240]. This event may explain another mechanism for FOXC2 dependent indirect inhibition of E-cadherin since *Zeb1* is a strong E-cadherin repressor. However, it seems that the effects of FOXC2 on cellular N-cadherin level is direct. Indeed, FOXC2 has been shown to directly bind to the *cdh2* promoter and induce its expression [241]. In concordance, inhibition of FOXC2 through a small-molecule inhibitor results in the reversal of cadherin switching and inhibition of metastasis [242]. Furthermore, inhibition of FOXC2 *in vivo* restores epithelial phenotype in metastatic Pca cells [237]. Therefore, it seems that FOXC2 plays a seminal role in establishing a positive loop among the transcription factors involved in cadherin switching. Indeed, SNAIL1 and TWIST1 can bind to the *FoxC2* promoter and increase its expression [235] feeding into the positive loop of cadherin switching.

### Sine oculis homeobox homolog 1 (Six1)

SIX1 is a homeobox transcription factor important in regulating genes involved in development. It is overexpressed in various cancers including PCa where it induces migration and invasion [243]. Although it is not yet clear whether the expression of *Six1* gene is directly regulated by androgen, enzalutamide-mediated inhibition of androgen signaling has been shown to increase *Six1* expression and promote castration resistance in PCa cells [244]. However, increasing evidence has been suggesting that this effect is not at the transcriptional level but is directly related to the increased protein stability of SIX1 [244]. In concordance, it was shown that triggering SIX1 degradation using a chemical inducer results in inhibition of cell growth and sensitization of PCa cells to enzalutamide-mediated castration [245]. In addition, increased SIX1 protein level causes a decrease in the E-cadherin and an increase in the N-cadherin levels [246,247]. However, the effects of SIX1 on E-cadherin and N-cadherin levels are probably regulated by indirect mechanisms [248]. Overexpression of *Six1* has been shown to promote tumor growth and metastasis in the breast cancer via both increasing TGF-β signaling and converting it from suppressive to supportive mode for tumor growth [246,249–251]. Six1 overexpression also promotes EMT in colorectal cancer through ZEB1 activation [251]. Furthermore, its overexpression promotes EMT and stem/progenitor cell phenotype in mammary tumor cells [252]. This tumor and EMT inducive role of *Six1* seems to be through increased levels of ZEB1, SNAIL1, and TWIST1 [250,251].

### SAM pointed domain containing ETS transcription factor (SPDEF)

SPDEF, also known as PDEF—prostate-derived ETS factor, is an androgen-regulated transcription factor that is dysregulated in many tumors. Contradicting results have been reported about its role in cancers. It has been reported to inhibit metastatic behaviors of PCa cells and its knockdown in mice results in increased metastasis [138]. Its metastasis-inhibiting role seems to be through inhibition of EMT via induction of epithelial/luminal phenotype [253]. However, a survey of more than 9,000 prostate tumor samples shows that expression of SPDEF is detected in 80% of these samples and its positivity is associated with the Gleason grade tumor stage and poor prognosis [254]. These contradicting reports might be related to the combination of other factors, tumor microenvironment, and the stages of the tumors.

ADT has been reported to decrease the SPDEF level that is associated with decreased *Cdh1* and increased *Cdh2* expression [255,256]. SPDEF can directly bind to the *Cdh1* promoter and promotes its expression [256]. However, it has been shown that this effect of SPDEF on *Cdh1* expression may be also regulated indirectly by controlling the *Twist1* expression, but independent of *Snail1* and *Snail2* activities [138,257]. In fact, SPDEF is considered a key molecule in the ADT-induced cadherin switching as its cellular level is directly controlled by AR and it directly regulates various cellular mechanisms [258]. For example, it is involved in the regulation of *Ccl2* expression, a chemokine involved in cadherin switching cooperatively via activating STAT3-TWIST1 signaling [257]. Consequently, SPDEF inhibits *Ccl2* expression and thereby cadherin switching in the presence of androgen, in PCa cells [255]. Conversely, ADT causes an increase in the *Ccl2* expression due to the inhibition of androgen signaling and subsequent repression of AR-SPDEF axis [255]. On the other hand, it was shown that ADT causes an increase in the TGF-βI level due to inhibition of the inhibitory AR-SPDEF axis [19]. Increased TGF-β signaling leads to increased expression of transcription factors *Zeb1*, *Snail1*, *Snail2*, and *Twist1*, leading to the induction of cadherin switching [19,137]. More studies is needed to elucidate the detailed role of SPDEF in the tumor progression and EMT in prostate cancer.

### N-myc downstream-regulated gene 1 (Ndrg1)

*Ndrg1* is an androgen-regulated gene, which is linked to the androgen network through interacting with β-catenin and heat shock protein 90 (HSP90) [259,260]. A recent study has shown that NDRG1 may regulate androgen signaling directly in PCa cells with important roles in both androgen-dependent and androgen-independent AR signaling [261]. NDRG1 protein is a suppressor of metastasis through inhibiting oncogenic TGF-β signaling [262–265]. Although NDRG1 protein is not a transcription factor, it has a crucial role in repressing the levels of transcription factors that induce cadherin switching, such as ZEB1, SNAIL1, and SNAIL2, through inhibition of TGF-β signaling [266,267]. In concordance, it has been shown that ADT causes a decrease in the *Ndrg1* expression, with a consequence of decreased *Cdh1* and increased *Cdh2* expressions [266]. Furthermore, ADT-induced increased N-cadherin inhibits the AR-NDRG1 axis in a c-Jun-dependent manner, creating a feedback loop that further induces *Cdh2* expression [266].

## Conclusion

Androgens have pivotal roles in the development and differentiation of the prostate gland. However, they also have critical roles in the pathogenesis of PCa. Although various treatment approaches are used for localized PCa tumors, ADT is the widely used standard treatment for advanced tumors. ADT is a powerful approach used to inhibit androgen signaling which leads to a successful response at the initial phase of treatment. However, androgen refractory tumors and metastases develop 24–36 months after ADT in many patients. Recent reports propose that ADT may also induce cadherin switching and ensuing metastasis in PCa. Indeed, growing pieces of evidence show that although ADT relieves the patients at the beginning of treatment, in advanced PCa, it may be the main cause for the development of metastasis via inducing cadherin switching.

ADT exerts a negative survival pressure on androgen-dependent PCa cells, inducing a homeostatic cellular response through activation of evolutionarily imprinted alternative survival mechanisms in cells, which may provide an advantage for growth of PCa cells with mutations leading to their androgen-independent survival. This is an evolutionary homeostatic response that in the long-range favors development of more aggressive CRPC. In this homeostatic response, decreased E-cadherin in the cells encourages anchorage independence and increased motility while increased N-cadherin encourages de-differentiation towards more stem cell-like characteristics in the cells. Meanwhile, other accumulated possible alterations, such as those leading to increased tonic signaling in the Wnt/β-catenin, TGF-β, PI3K/AKT, MAPK, and other pathways, fortify this transition leading to more aggressive and treatment-resistant CRPC. Developing approaches employing combination treatment strategies, such as targeting motility and survival, along with ADT to help prevent survival of initial mutants that will otherwise develop into CRPC may prove beneficial.
